# miR-4482 and miR-3912 aim for 3ʹUTR of ERG mRNA in prostate cancer

**DOI:** 10.1371/journal.pone.0286996

**Published:** 2023-06-13

**Authors:** Sidra Mumtaz, Muhammad Usman Rashid, Rizwan Ullah Khan, Naila Malkani

**Affiliations:** 1 Department of Zoology, GC University, Lahore, Pakistan; 2 Department of Basic Sciences Research, Shaukat Khanum Memorial Cancer Hospital and Research Centre (SKMCH&RC), Lahore, Pakistan; Northwest University, UNITED STATES

## Abstract

Ets-related gene (ERG) is overexpressed as a fusion protein in prostate cancer. During metastasis, the pathological role of ERG is associated with cell proliferation, invasion, and angiogenesis. Here, we hypothesized that miRNAs regulate ERG expression through its 3ʹUTR. Several bioinformatics tools were used to identify miRNAs and their binding sites on 3ʹUTR of ERG. The selected miRNAs expression was analyzed in prostate cancer samples by qPCR. The miRNAs overexpression was induced in prostate cancer cells (VCaP) to analyze ERG expression. Reporter gene assay was performed to evaluate the ERG activity in response to selected miRNAs. The expression of ERG downstream target genes was also investigated through qPCR after miRNAs overexpression. To observe the effects of selected miRNAs on cell proliferation and migration, scratch assay was performed to calculate the cell migration rate. miR-4482 and miR-3912 were selected from bioinformatics databases. miR-4482 and -3912 expression were decreased in prostate cancer samples, as compared to controls (*p<0*.*05* and *p<0*.*001*), respectively. Overexpression of miR-4482 and miR-3912 significantly reduced ERG mRNA (*p<0*.*001* and *p<0*.*01*), *respectively*) and protein (*p<0*.*01*) in prostate cancer cells. The transcriptional activity of ERG was significantly reduced (*p<0*.*01*) in response to miR-4482 and-3912. ERG angiogenic targets and cell migration rate was also reduced significantly (*p<0*.*001*) after miR-4482 and -3912 over-expression. This study indicates that miR-4482 and -3912 can suppress the ERG expression and its target genes, thereby, halt prostate cancer progression. These miRNAs may be employed as a potential therapeutic target for the miRNA-based therapy against prostate cancer.

## Introduction

ETS-related gene (ERG) is a member of the E-26 transformation specific (ETS) family of transcription factors. It was mapped on chromosome 21 (21q22.2). It encodes a 54 kDa (kilo Dalton) protein (P11308) [1]. ETS proteins are nuclear DNA-binding phosphoproteins that act as activators or repressors of transcription [2]. ERG is expressed in endothelial cells (ECs) [3], particularly in the blood vessels surrounding the neural tube [4], heart vasculature, and the pre-cartilage [5]. It plays a key role in the regulation of tissue-specific processes such as haematopoiesis, vascular inflammation, and angiogenesis [6], by regulating the WNT/β-catenin signaling pathway and the transcriptional control of EC-specific genes including angiopoietin 2, endoglin, von Willebrand factor (vWF), VEGF-A [7] and VE-cadherin [8]. ERG expression increases in various carcinomas and leads to angiogenesis and primary drug resistance [9].

Prostate cancer is the second most common cancer among elderly males worldwide, accounting for 1,414,259 new cases and 375,304 deaths in 2020 [10]. It is a recurrent cancer with high mortality among males worldwide [11]. The majority of these patients are diagnosed with elevated levels of prostate-specific antigen. However, a tissue biopsy is required to confirm this malignancy [10]. The treatment strategy of this cancer includes surveillance, prostatectomy, radiotherapy and hormone therapy [12]. The role of ERG in prostate cancer is well characterized. It is involved in prostate intraepithelial neoplasia, invasion, and metastasis [13]. The up-regulation of ERG is observed in >50% of prostate cancer cases, due to a genetic fusion between trans-membrane serine protease 2 (TMPRSS2) and ERG [14]. This gene fusion activates the ERG oncogenic pathway, which contributes to the disease progression. The ERG rearrangements are reported in 23 of 29 prostate cancer samples. Cell culture experiments revealed that the TMPRSS2 androgen-responsive promoter elements mediate the overexpression of ETS in prostate cancer [13].

Altered gene expression is crucial for the maintenance of a disease condition as it changes the overall cellular functions. Gene expression is affected in the cell due to several post-transcriptional modifications. 3ʹ untranslated regions (3ʹUTR) are the mRNA sites that have regulatory elements such as microRNA response elements (MREs), AU-rich elements (AREs), and poly(A) tail [15]. MREs are the sequences where microRNAs (miRNA) bind through their seed sequences and result in the mRNA translational repression. miRNAs are small non-coding RNAs involved in regulating several physiological conditions. The consequences of miRNA binding to 3ʹUTR are the transformed biological pathways resulting in the proliferation, differentiation, and apoptosis of cells [16]. However, miRNA expression dysregulation has been reported in cancers due to deletion, insertion, translocation, or amplification of miRNA genes, loss or defects in miRNA biogenesis, and epigenetic changes. This results in aberrant expression of target gene mRNA which in return causes abnormal cellular processes leading to disease progression [17].

In prostate cancer, several miRNAs regulate the ERG expression such as miR-9 [18], miR-26, miR-182, miR-200b [19], and miR-221 [20]. The change in ERG expression due to these miRNAs leads to irregular apoptosis and cell proliferation. In the current study, we employed bioinformatics prediction tools to identify the potential miRNAs against the 3ʹUTR of ERG. The identified miRNAs were tested in various assays to understand their effects on the ERG expression and targeted processes.

## Material and methods

### Selection of miRNAs for ERG mRNA

To predict miRNAs binding to 3ʹUTR of ERG, miRDB (http://www.mirdb.org/cgi-bin/search_custom.cgi), miRBase (http://www.mirbase.org/), TargetScan (http://www.targetscan.org/vert_72/) and miRWalk (http://zmf.umm.uni-heidelberg.de/apps/zmf/mirwalk2/) were used. The miRNAs with the highest prediction score (≥ 95) and minimum free energy (MFE) (≈ -25KJ/mol) were selected.

### Acquisition of prostate cancer samples and controls

Formalin-fixed paraffin-embedded (FFPE) tissues of prostate cancer samples (n = 25) and controls (n = 20) were collected in thin slices (20μm micron) from the department of pathology, Shaukat Khanum Memorial Cancer Hospital and Research Centre (SKMCH&RC), Lahore, Pakistan. The tissue adjacent to the prostate tumor without any malignancy was considered as control. A qualified pathologist reviewed all slides to identify prostate cancer foci and adjacent normal glandular epithelium.

### Ethical approval and consent

The study was approved by Institutional Review Board (IRB) of the SKMCH&RC, Lahore, Pakistan via letter No. EX-05-03-19-04. As the study was retrospective and patient identity was kept confidential by the hospital therefore no verbal or written consent was required.

### Cell culture

Epithelial cells of human prostate cancer (VCaP) were cultured and propagated in Dulbecco’s modified MEM medium (DMEM) (Cat#D5648, Sigma-Aldrich, Taufkirchen, Germany) supplemented with penicillin (100 units/mL), streptomycin (100 μg/mL) (Cat#P4333, Sigma-Aldrich, Taufkirchen, Germany), and 10% fetal bovine serum (FBS) (Cat#FBS-22A, Capricorn scientific, Ebsdorfergrund, Germany) with 5% CO_2_ at 37°C. Human Vascular Endothelial Cells (HUVEC) were isolated and cultured in M199 medium having 10%FBS and endothelial growth factors.

### Expression plasmids and transfections

The expression plasmids of miR-4482 and miR-3912 were constructed from the seed sequences (miR-4482: UUUCUAUUUCUCAGUGGGGCUC; miR-3912: AUGUCCAUAUUAUGGGUUAGU) in the pEGFP-C1 vector (6084–1, Clontech, USA Takara Bio USA, Inc). Following primers were used to PCR amplify from human genomic DNA.

miR-4482-F: 5’-TCGA*AAGCTT*GCTGAATCGGAAATGCAGCG-3’ (*HindIII*)
miR-4482-R: 5’-AGCT*GGATCC*TCCTCCTTCTTTCATTTCC-3’ (*BamHI*)
miR-3912-F: 5’-TCGA*CTCGAG*CATTCCTCCTGAAAGAAAGC-3’ (*XhoI*)
miR-3912-R: 5’-AGTTGGATCCCAGCACTTTGGAAGGCAGAG-3’ (*BamHI*)

These primers with specific restriction sites were designed by retrieving sequences of precursor miRNA from Ensembl (http://ensembl.org). The amplified PCR product resulted into the inserts of 618 bp and 625 bp for miR-4482 and miR-3912, respectively. The product was gel purified, followed by restriction-enzyme digestion of inserts as well as CMV-eGFP vector and ligation. The ligation mixture was transformed into DH5α cells (chemically competent) and the resultant clones were tested by restriction analysis and sequencing. The positive clones were then selected for further experiments.

1kb of 3ʹUTR (GenBank accession number NG_029732) of wild-type ERG cloned into pMIR-RNL-TK was generously provided by Prof. Sven Wach [21]. All transient transfections were carried out using Turbofect (Cat#R0531, Thermoscientific, USA) according to manufacturer’s protocol.

### Quantitative real-time PCR

Quantitative real-time PCR (qRT-PCR) was performed to identify the mRNA expression of ERG, its downstream target genes, and miRNA. Appropriate plasmids were transfected into VCaP or HUVEC, harvested after 48h and total RNA were isolated using TRIzol [22]. Similarly, the FFPE samples of prostate cancer and controls were proceeded for RNA extraction using the method described by Ma et al., 2009 [23]. 1μg RNA was converted to cDNA from each sample using the cDNA synthesis kit (Cat#K1622, Thermoscientific, USA; Cat#G898, Applied Biological Materials, USA), according to the manufacturer’s protocols. qPCR assay was performed in triplicates using 1μl of each cDNA and a SYBR green master mix (Cat#K0222, Thermo Fisher scientific, USA). The data was processed in LinRegPCR software [24] and the Pfaffl equation [25] was used for calculations. U6 and GAPDH were used for data normalization where appropriate. Following primer sequence were used:

miR-4482: Forward: 5’—GTTGGGTTTCTATTTCTCAGTG - 3’
       Reverse: 5’-GTGCAGGGTCCGAGGT-3’
miR-3912: Forward: 5’-GTTTGGGATGTCCATATTATGG-3’
       Reverse: 5’-GTGCAGGGTCCGAGGT-3’
U6: Forward: 5’-CTCGCTTCGGCAGCACA-3’
    Reverse: 5’-GTGCAGGGTCCGAGGT-3’
GAPDH: Forward: 5’-CCTGTTCGACAGTCAGCCG-3’
        Reverse: 5’-CGACCAAATCCGTTGACTCC-3’
ERG: Forward: 5’-AACGAGCGCAGAGTTATCG-3’
     Reverse: 5’-GTGAGCCTCTGGAAGTCGTC-3’
VE-cad: Forward: 5’-TGTGGGCTCTCTGTTTGTTG-3’
      Reverse: 5’-AATGACCTGGGCTGTGTTTC-3’
FZD4: Forward: 5’-CCTCGGCTACAACGTGACC-3’
      Reverse: 5’-TGCACATTGGCACATAAACAGA-3’
EGFL7: Forward: 5’-TGAATGCAGTGCTAGGAGGG-3’
      Reverse: 5’-GCACACAGAGTGTACCGTCT-3’
VEGF: Forward: 5’-AGGGCAGAATCATCACGAAGT-3’
     Reverse: 5’-TGCCATCCAATCGAGACCCT-3’

### Western blotting

VCaPs were seeded in 6-well plate (2x10^5^ cells/well), transfected with miR-4482 (2 μg/well) and miR-3912 (2 μg/well) plasmids using turbofect (5 μl/well). Western blotting was performed to analyze the ERG protein expression. Briefly, after 48 h of transfection, cells were lysed in RIPA buffer on ice followed by centrifugation (14,000g for 15 minutes at 4ºC). Total protein concentration in each sample was measured by Bradford method [26]. Equal amount of total proteins from each sample were loaded onto mini-gels for SDS-PAGE, followed by blotting and detection. ERG primary antibody (Cat#97249, Cell Signaling Technology, Danvers, USA) and Anti-rabbit IgG, HRP linked secondary antibody (Cat#7074, Cell Signaling Technology, Danvers, USA) were used at the final dilution of 1:1000 and 1:10,000, respectively. The blots were visualized by enhanced chemiluminescence, and quantified by ImageJ. β-actin (Cat#8459, Cell Signaling Technology, Danvers, USA) was used as a loading control [27].

### Luciferase reporter assay

Reporter gene assay was performed to assess the transcription activity of ERG as a result of miRNAs binding. VCaP cells were transfected in 12-well plates (1x10^5^ cells/well) with a reporter plasmid (ERG3ʹUTR-pMIR-RNL-TK), β-galactosidase and miR-4482 or miR-3912 plasmids in triplicates. The cells were lysed after 24 h of transfection and centrifuged at 14,000rpm for 20 minutes. The supernatant (20 μl) was mixed with assay buffer (50 μl) and luciferin (50 μl) in 96-well plate and luminescence were measured. After measurement of luminescence 50 μl of Chlorophenol-red β-d-galactopyranoside (CPRG) (Cat#59767, Sigma-Aldrich, Taufkirchen, Germany) substrate was added in each well, incubated for 2 minutes and measured at 595 nm. Change in CPRG color from yellow to orange/red indicated the activity of β -galactosidase which was used to normalize the luciferase activity [28].

### Scratch assay

The scratch assay was performed to analyze the cell migration. HUVEC were seeded in 12-wells and transfected with miR-4482, miR-3912, and pEGFP-C1 control vectors. A scratch was made in each well with a sterile 20 μl micropipette tip. At the 4 h interval, scratch closure was observed till 48 h. The cell migration was evaluated as the percentage of scratch closure by measuring the difference of cell-free surface of the scratch at various time intervals using ImageJ [27].

## Results

### miRNAs bind to 3ʹUTR of ERG

**Fig 1:**
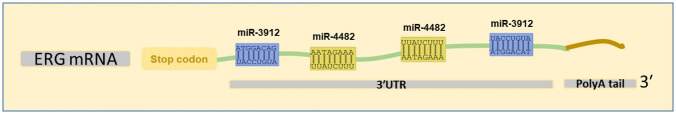
Binding of miR-4482 (-372+, - 445+), and miR-3912(-214+, -565+) at 3′UTR of ERG (3381bp).

The *miRDB* and *TargetScan* database analyses showed that the 3′UTR of ERG is a highly ranked potential target of miR-4482 and miR-3912. miR-4482 has two binding sites with a 96-target score and MFE of -25.8KJ/mol. miR-3912 also binds on two sites at 3′UTR with a 95-target score and MFE of -23.5KJ/mol (Fig 1). The *miRBase* and *miRwalk* databases did not predict miR-3912 binding to the 3′UTR of ERG. We further explored these miRNAs due to the strong prediction score of miR-4482 and miR-3912 for ERG in the first two databases. A list of other miRNAs predicted by these databases is provided in S1 Table.

### Expression of miR-4482 and miR-3912 in prostate cancer

**Fig 2:**
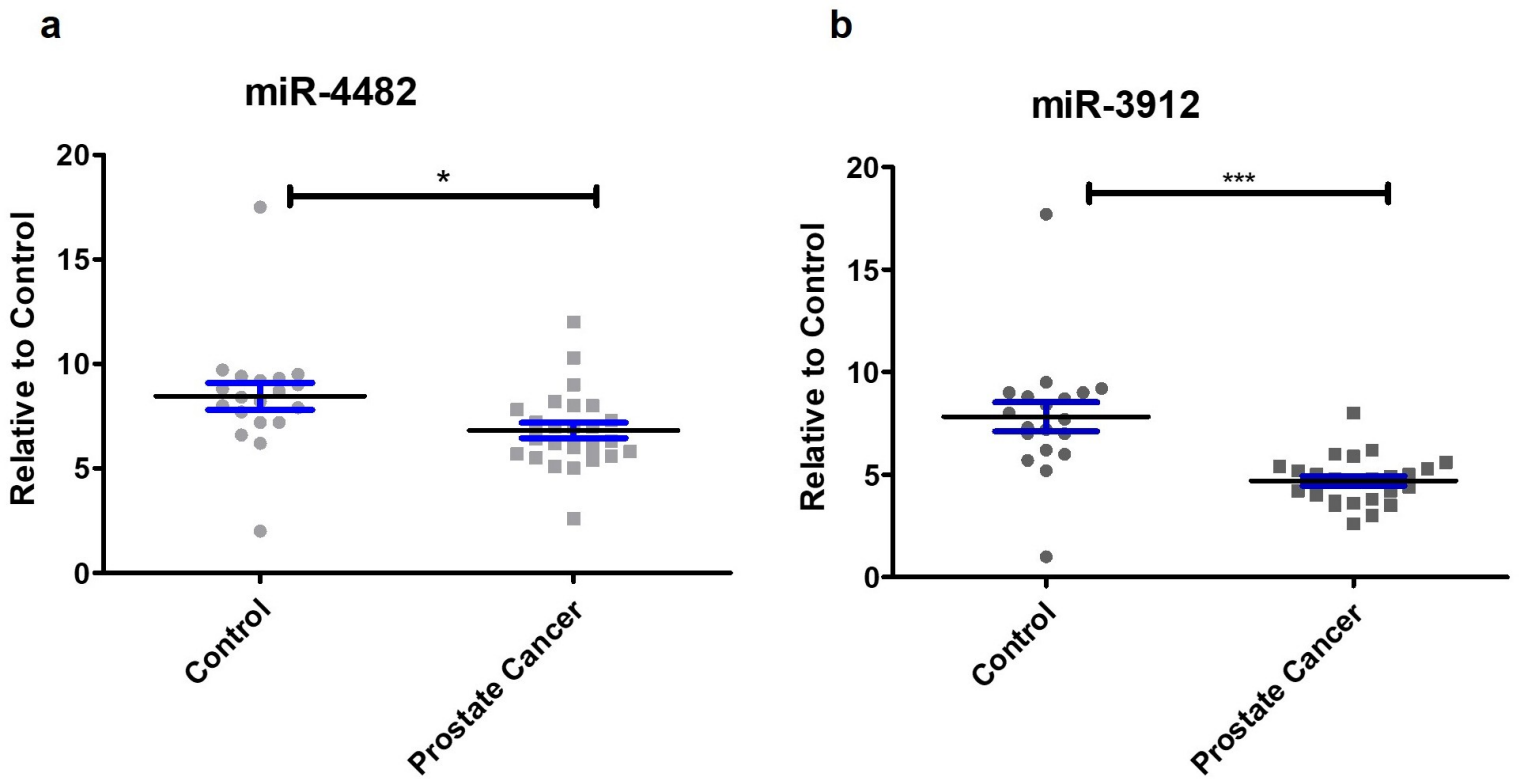
Expression of miR-4482 (a) and miR-3912 (b) in prostate cancer samples with Gleason score 4/5. Data expressed as mean ± SEM, *p < 0.05; ***p < 0.001.

The miR-4482 and miR-3912 expression was significantly reduced in prostate cancer samples as compared to controls (*p* < 0.001 and *p* < 0.05), respectively. All patients with prostate cancer presented with Gleason score of 4/5 (Fig 2a and 2b).

### Ectopic expression of selected miRs influences ERG expression

**Fig 3:**
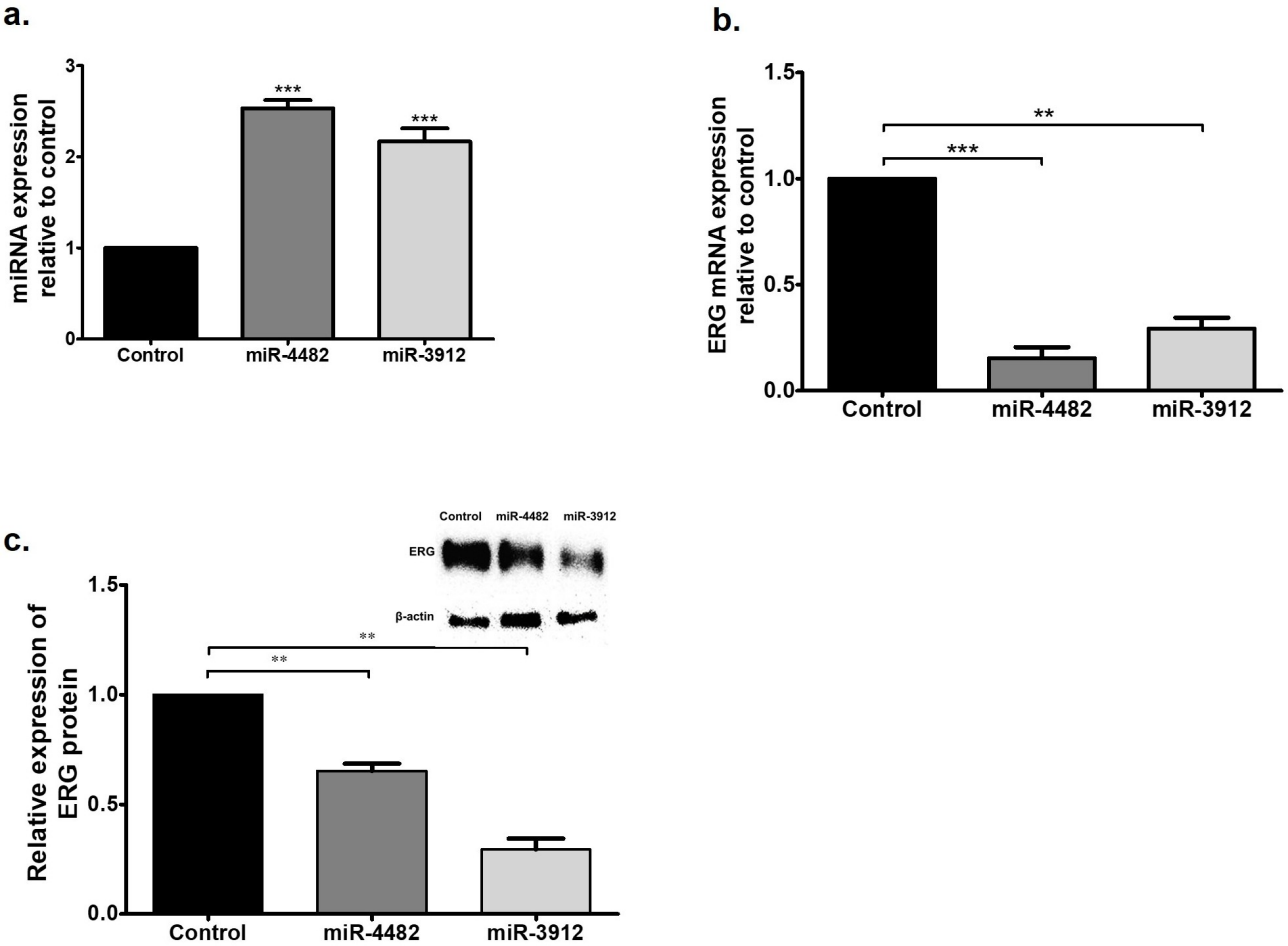
Ectopic expression of miR-4482 and miR-3912 (a) reduced ERG mRNA expression relative to GAPDH (b) and ERG Protein expression relative to β-actin (c). Data expressed as mean ± SEM, **p < 0.01; ***p < 0.001.

VCaPs were separately transfected with miR-4482 and miR-3912, and the ERG expression was analyzed 48 h after transfection in comparison to control cells. The ERG expression of ERG mRNA and protein was significantly reduced (*p* < 0.001 and *p* < 0.01) in response to miR-4482 and miR-3912, respectively (Fig 3a and 3b).

### miRs regulates ERG transcription at 3′UTR

**Fig 4:**
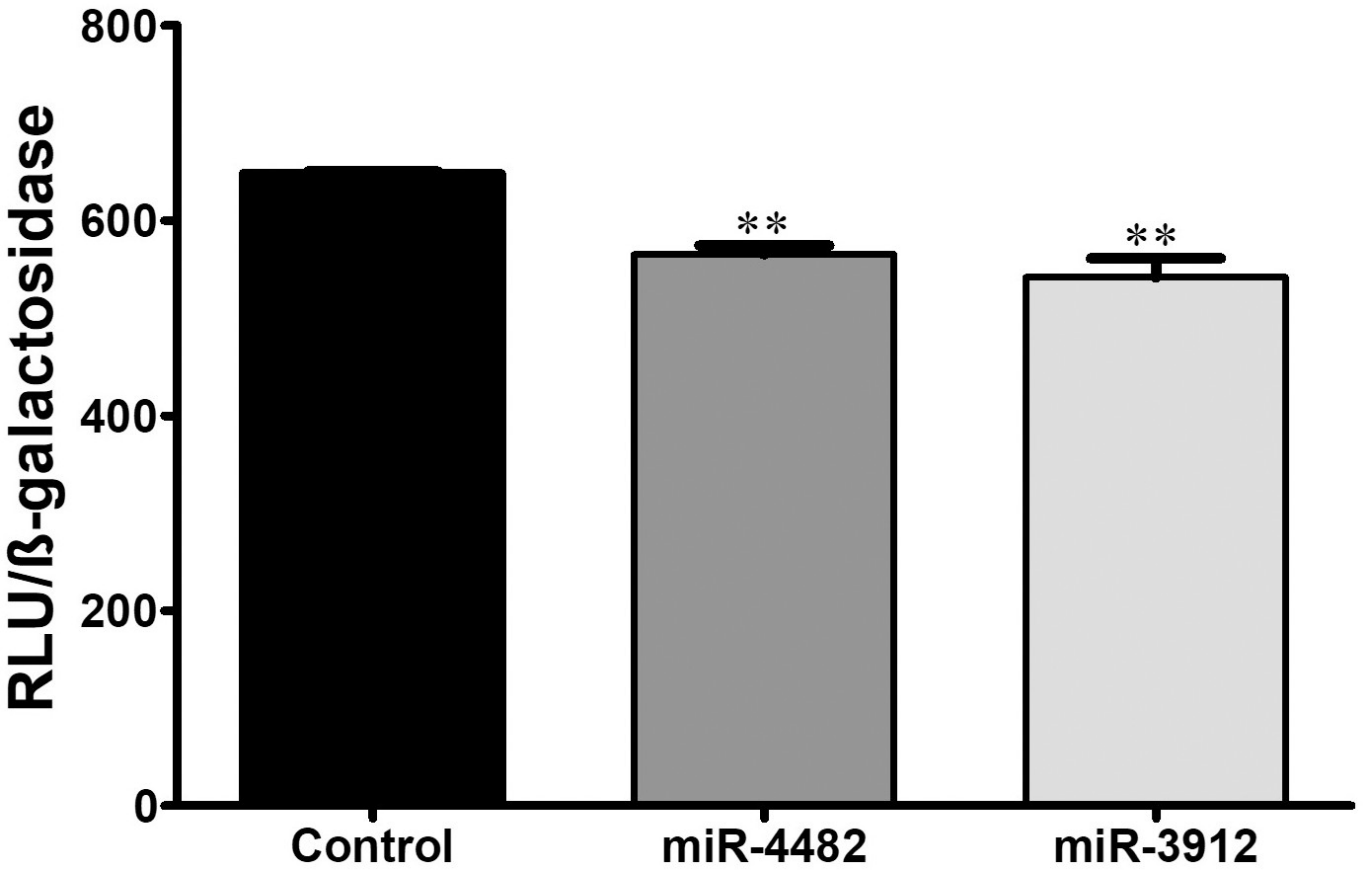
Luciferase reporter gene assay in VCaP cells with 3′UTR of ERG co-expressed with miR-4482 or miR-3912 showed reduced transcriptional activity of ERG. n = 3. Data expressed as mean ± SEM, **p < 0.01.

To determine whether miR-4482 and miR-3912 binding on the 3′UTR of ERG affect the transcription activity of ERG, a luciferase reporter assay was performed with pMIR-RNL-TK-3′UTR ERG, co-expressed with β-galactosidase plasmid and miR-4482 or miR-3912 in VCaP cells. Cells transfected with miR-4482 or miR-3912 showed significantly reduced reporter gene activity (*p* < 0.01). Appropriate transfection control was used for comparison (Fig 4).

### Ectopic expression of miRs affect ERG target genes

**Fig 5:**
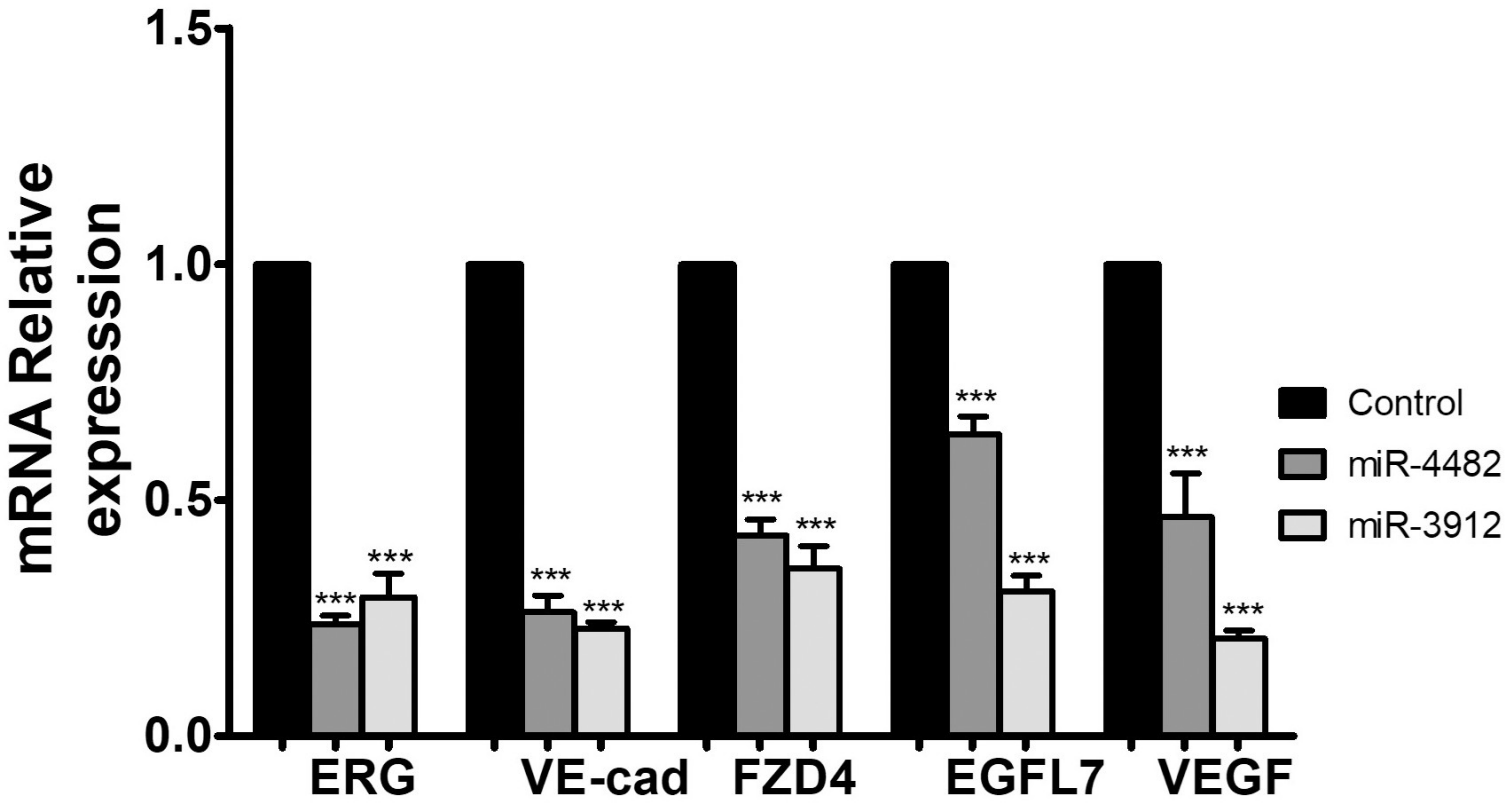
mRNA expression of ERG targets genes altered in miR-4482 and miR-3912 transfected HUVEC. Data expressed as mean ± SEM, *** p < 0.001.

To determine the ERG target genes expression, HUVEC were transfected with miR-4482 and miR-3912 using electroporation [29]. After 48 h cells were lysed and proceeded for mRNA detection through qPCR. The ERG target genes were significantly reduced in miRs transfected cells as compared to control cells (*p*<0.001). GAPDH was used for normalization (Fig 5).

### miRs mediated suppression of cell migration

**Fig 6:**
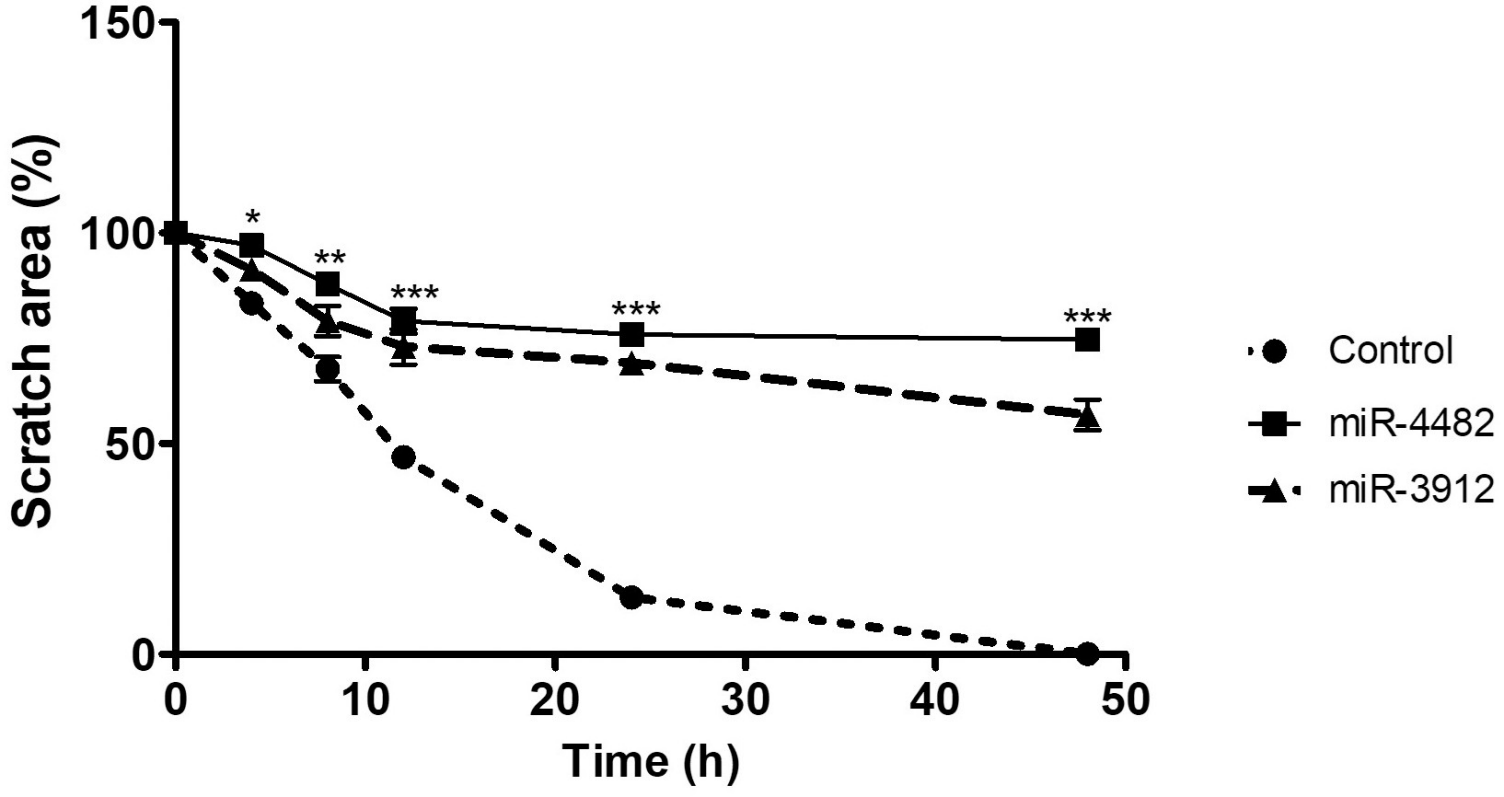
HUVEC transfected with miR-4482 and miR-3912 reduced the migration of cells. n = 3 Data expressed as percentage of cell free area. Data expressed as mean ± SEM, *p < 0.05, **p < 0.01, *** *p* < 0.001.

A scratch assay was performed to evaluate the miR-4482 and miR-3912 effect on cell migration. HUVEC were independently transfected with miR-4482 and miR-3912. miRs transfected cells showed reduced migration rate in comparison to control cells (GFP transfected). Cell migration and proliferation were observed for 48 h after applying the scratch. In control cells, the cell-free area was closed within 48 h, while in miR-4482 and miR-3912 transfected cells a gap was still visible after 48 h. The cell-free area was quantified over time and expressed as a percentage of the initial scratch area (Fig 6).

## Discussion

ERG is a transcription factor with diverse functions in endothelium and several malignancies including prostate cancer. In prostate cancer, ERG overexpression leads to ductal dysplasia, PIN [30] metastasis, and advanced disease which may be related to the role of ERG in promoting angiogenesis. The ERG overexpression leads to increased cell proliferation, and invasion. ERG also modifies prostate epithelium by repressing epithelium-specific genes such as Prostein and Prostate Specific Antigen [31]. The molecular crosstalk between ERG and androgen receptor (AR) has implications in the complex network of prostate cancer signaling pathways [32]. Based on these varied consequences of ERG overexpression, it is logical to use therapeutic agents that can target ERG in prostate cancer. In this regard, miRNAs can present significant prospects. Previously, miR-145 and miR-196 expressions are shown to downregulate ERG expression in prostate cancer [33] and leukemia, respectively [20]. In the current study, we have identified two miRNAs (miR-4482 and miR-3912) which efficiently targeted the 3ʹUTR of ERG and resulted in the decreased expression of ERG mRNA and protein. ERG downstream gene targets expression was also decreased and hence the transcription and migration functions related to ERG were modulated in response to the miRs binding. These results showed that ERG function was modulated as miRs bind to its mRNA, thereby supporting the notion that these miRNAs can be used as therapeutic agents to modulate ERG functions in prostate cancer.

In the current study, miR-4482 and miR-3912 expression in prostate cancer samples was significantly decreased as compared to the controls. The bioinformatics databases revealed that both these miRNAs have high potential to bind to the 3ʹUTR of ERG mRNA. *In-vitro* assays further confirmed that this binding influences ERG expression. Moreover, ERG mRNA and protein were significantly reduced in prostate cancer cells (VCaP). The reduced transcription activity of ERG as a result might help to reduce inflammation in the prostate. The downregulation of ERG influenced the ERG functions by downregulating its target gene expression involved in cell migration and adhesion (Fig 5).

miR-4482 has been previously reported to have differential expression in several diseases such as pediatric embryonal central nervous system neoplasms [34], immune thrombocytopenia [35, 36], and infections [37]. Similarly, miR-3912 is differentially expressed in papillary thyroid cancer patients [38] and pediatric embryonal central nervous system neoplasms [34]. It is shown to bind to 3ʹUTR of NEIL2 (Nei Like DNA Glycosylase 2) to contribute to age related cataracts by interfering with the DNA repair mechanism [39]. Moreover, in esophageal squamous cell carcinoma RALYL (RALY RNA Binding Protein-like) is targeted by miR-3912 to hinder transcriptional and post-transcriptional regulatory processes [40].

In this study, we have shown that miR-4482 and miR-3912 negatively influence the cell migration potential by targeting 3ʹUTR of ERG. Also, the angiogenic target genes of ERG are also down-regulated in response to the over-expression of these miRs. Angiogenesis is a crucial step leading to metastasis and ERG regulates this process by regulating the expression of Ve-cadherin, eNOS, and VEGF [41]. It stabilizes the vasculature and ensures nutrient supply to the growing tumor. ERG inhibition reduced the expression of VE-cadherin and EGFL7 that may result in decreased proliferation and increased apoptosis due to junction instability, hence, inhibit tumor metastasis. Moreover, the downregulation of vascular endothelial growth factor (VEGF), a mediator of angiogenesis, and Frizzled Class Receptor 4 (FZD4), may also contribute to further reducing invasiveness and cancer progression.

miRNAs’ potential to act as therapeutic agents should be further explored as their cellular expression can be regulated to adjust the expression of their gene targets. Several miRNAs are undergoing clinical trials to be designated as anticancer drugs [42]. miR-145 is associated to prostate cancer progression [43] and a therapeutic molecule (MGN-2677) based on miR-145 cluster is in preclinical trials [42]. miR-29b is also known to involve in the suppression of prostate cancer metastasis [44]. The miRNAs regulating ERG expression should be explored and tested for their drug potential in those conditions where aberrant ERG expression of is a major cause. The miR-4482 and miR-3912 can bind to ERG mRNA at definite sequences in 3ʹUTR and may act as potential tumor suppressors.

## Conclusion

In conclusion this is the first study investigating that miR-4482 and miR-3912 suppress the ERG expression and its target genes, and thereby halt the prostate cancer progression. 3ʹUTR of ERG plays a significant role in the modulation of ERG expression and function in prostate cancer by providing binding sites to miRNAs. miR-4482 and miR-3912 should be further investigated as potential therapeutic targets for prostate cancer.

## Supporting information

S1 Table List of predicted miRNAs. (DOCX)

S1 Raw images (PDF)
