# Supplementary material for: miR-4482 and miR-3912 aim for 3ʹUTR of ERG mRNA in prostate cancer
Source: PLoS One. 2023 Jun 13;18(6):e0286996. doi: 10.1371/journal.pone.0286996 (PMC10263311; doi:10.1371/journal.pone.0286996)
Supplement: S1 Table — (DOCX) [file pone.0286996.s001.docx]

| **miRNA** | **Sequence** | **MFE kj/mol** | **Score** |
| --- | --- | --- | --- |
| hsa-miR-4482-3p | UUUCUAUUUCUCAGUGGGGCUC | -26.9 | 96 |
| hsa-miR-3912-5p | AUGUCCAUAUUAUGGGUUAGU | -23.5 | 95 |
| hsa-miR-361-3P | UCCCCCAGGUGUGAUUCUGAUUU | -27.4 | 96 |
| hsa-miR-4717-5p | UAGGCCACAGCCACCCAUGUGU | -29.2 | 90 |
| hsa-miR-3690 | ACCUGGACCCAGCGUAGACAAAG | -29.6 | 89 |
| hsa-miR-137 | UUAUUGCUUAAGAAUACGCGUAG | -19.8 | 85 |
| hsa-miR-4445-3p | CACGGCAAAAGAAACAAUCCA | -20.1 | 78 |
| hsa-miR-4445-5p | AGAUUGUUUCUUUUGCCGUGCA | -29.1 | 77 |
| hsa-miR-3670 | AGAGCUCACAGCUGUCCUUCUCUA | -32.4 | 73 |

Supp.Table 1: List of predicted miRNAs
