# Supplementary figures and images for: miR-4482 and miR-3912 aim for 3ʹUTR of ERG mRNA in prostate cancer
Source: PLoS One. 2023 Jun 13;18(6):e0286996. doi: 10.1371/journal.pone.0286996 (PMC10263311; doi:10.1371/journal.pone.0286996)

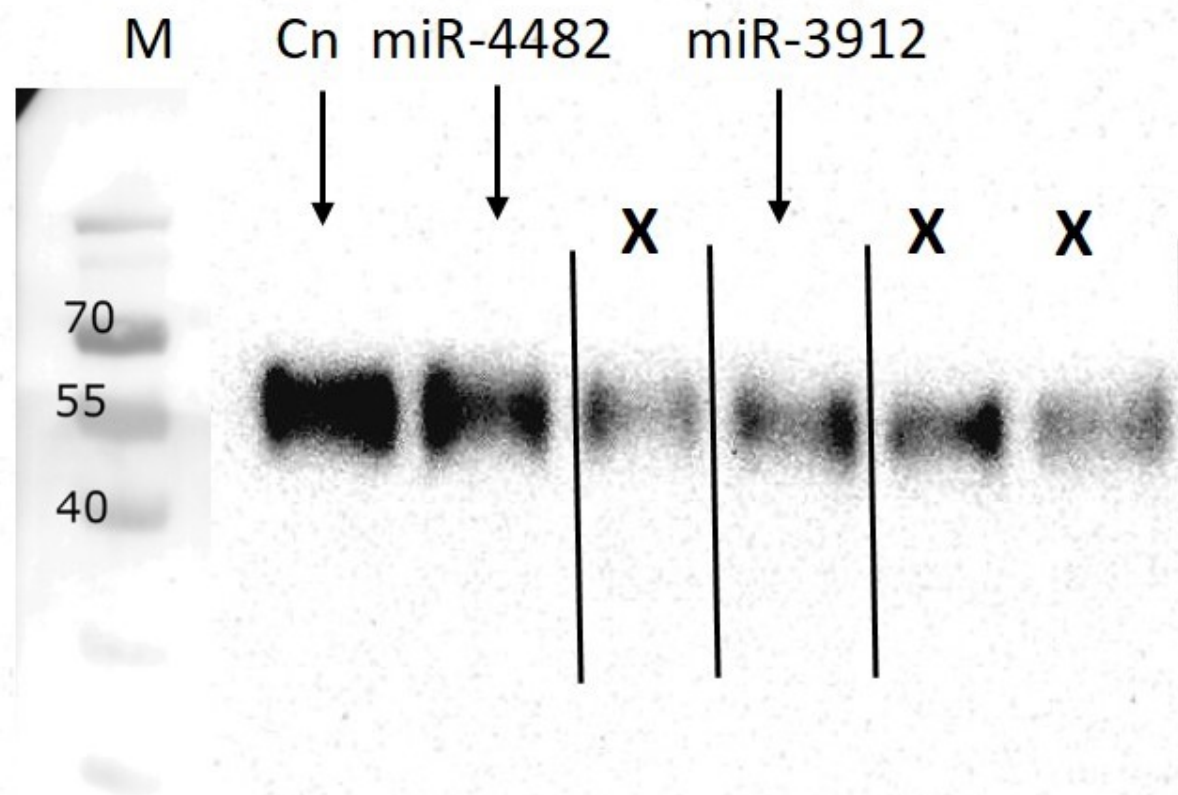

ERG for figure 3c

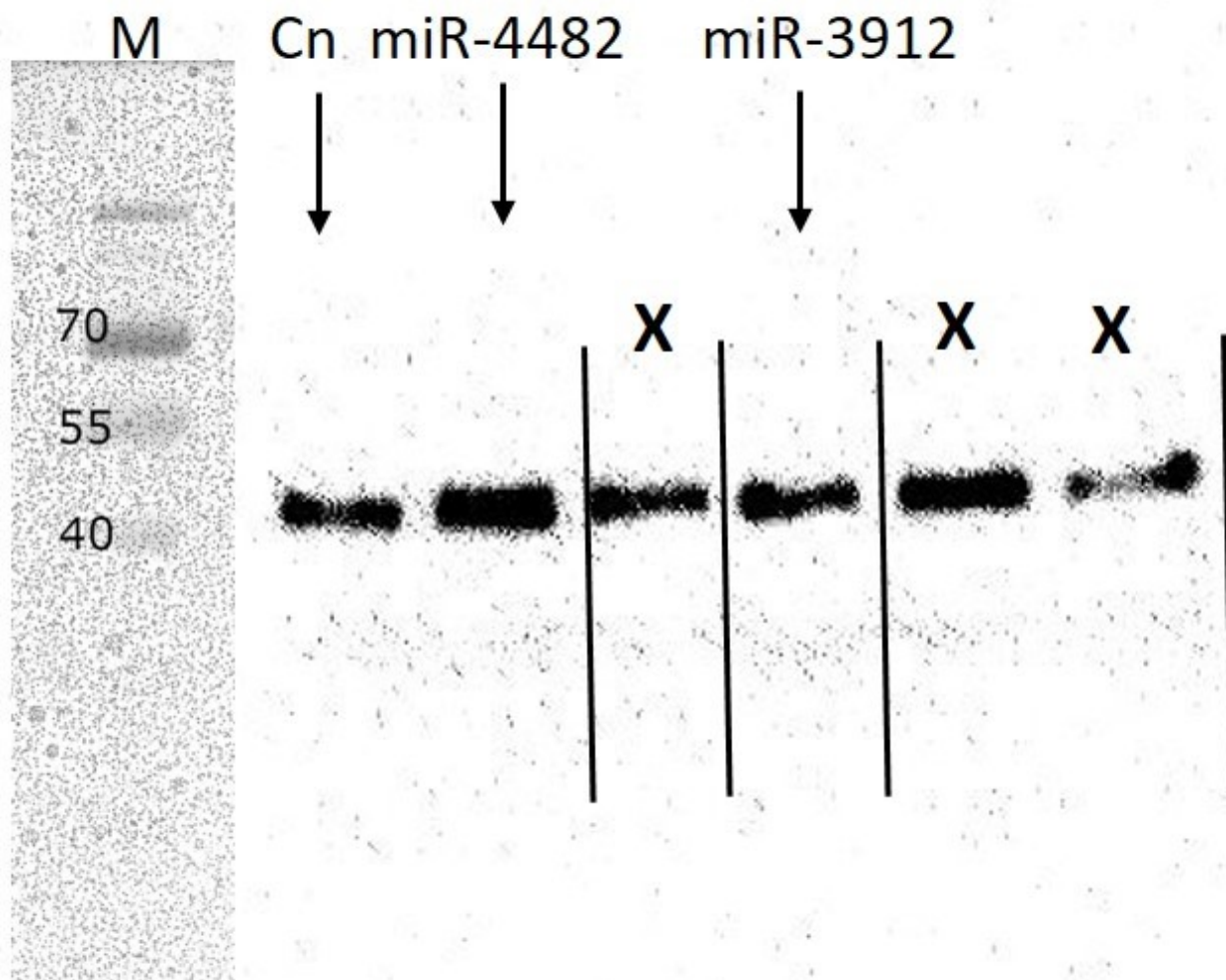

Actin control for figure 3c

Supplement: S1 Raw images — (PDF) [file pone.0286996.s002.pdf]
